# Evaluating the Diagnostic Value of Electrovestibulography (EVestG) in Alzheimer’s Patients with Mixed Pathology: A Pilot Study

**DOI:** 10.3390/medicina59122091

**Published:** 2023-11-28

**Authors:** Zeinab A. Dastgheib, Brian J. Lithgow, Zahra K. Moussavi

**Affiliations:** Diagnostic and Neurological Processing Research Laboratory, Biomedical Engineering Program, University of Manitoba, Riverview Health Centre, Winnipeg, MB R3L 2P4, Canada; zeinab.dastgheib@umanitoba.ca (Z.A.D.); brian.lithgow@umanitoba.ca (B.J.L.)

**Keywords:** feature selection, diagnostic algorithm, Electrovestibulography, Alzheimer’s disease, cerebrovascular pathology, gamma-aminobutyric acid

## Abstract

*Background and Objectives*: Diagnosis of dementia subtypes caused by different brain pathophysiologies, particularly Alzheimer’s disease (AD) from AD mixed with levels of cerebrovascular disease (CVD) symptomology (AD-CVD), is challenging due to overlapping symptoms. In this pilot study, the potential of Electrovestibulography (EVestG) for identifying AD, AD-CVD, and healthy control populations was investigated. *Materials and Methods*: A novel hierarchical multiclass diagnostic algorithm based on the outcomes of its lower levels of binary classifications was developed using data of 16 patients with AD, 13 with AD-CVD, and 24 healthy age-matched controls, and then evaluated on a blind testing dataset made up of a new population of 12 patients diagnosed with AD, 9 with AD-CVD, and 8 healthy controls. Multivariate analysis was run to test the between population differences while controlling for sex and age covariates. *Results*: The accuracies of the multiclass diagnostic algorithm were found to be 85.7% and 79.6% for the training and blind testing datasets, respectively. While a statistically significant difference was found between the populations after accounting for sex and age, no significant effect was found for sex or age covariates. The best characteristic EVestG features were extracted from the upright sitting and supine up/down stimulus responses. *Conclusions*: Two EVestG movements (stimuli) and their most informative features that are best selective of the above-populations’ separations were identified, and a hierarchy diagnostic algorithm was developed for three-way classification. Given that the two stimuli predominantly stimulate the otholithic organs, physiological and experimental evidence supportive of the results are presented. Disruptions of inhibition associated with GABAergic activity might be responsible for the changes in the EVestG features.

## 1. Introduction

Dementia is a progressive clinical syndrome, describing an array of brain disorders with debilitating cognitive and behavioral impairments [1]. Diagnosis of dementia is based on clinical symptoms, i.e., medical history, neuropsychiatric and neuropsychological assessments as well as brain imaging results and genetic tests [1,2]. Alzheimer’s disease (AD) and vascular dementia (VaD) are the most common types of dementia, and make up to around 60% and 30% of all cases, respectively [3]. Given that the chance to develop cerebrovascular disease (CVD) increases with age [4], AD patients often present with varying levels of CVD symptomology, and are considered as a dementia subtype called AD-CVD [5,6,7]. Differential diagnosis of AD and AD-CVD is challenging due to overlapping symptomologies [1,2]. Currently, brain autopsy is the only way to confirm dementia and its subtypes [8].

AD and AD-CVD have commonalities but also differences in their characteristics, which may both hinder and help their separation. Given that the AD-CVD pathology sits in the continuous spectrum between AD and VaD, and due to the involvement of cerebrovascular pathology, i.e., cerebrovascular lesions/blood flow reductions, AD-CVD has been found to be associated with a more rapid cognitive decline that often ends in a more severe form of dementia than predominant AD pathology [9,10]. On the other hand, AD-CVD pathology has been associated with a lower burden of Amyloid-β (Aβ) pathology than dementia with AD predominance [11,12], suggesting the presence of less AD pathology in AD-CVD compared to that in AD patients [13,14]. Regardless of the difference in origins, AD and AD-CVD both demonstrate neurodegenerative pathology, which makes their distinction complex due to similar symptoms, specifically, at early stages of the disease. While there are accepted criteria to diagnose AD and VaD [2,6,15], there is little consensus for the diagnosis of AD-CVD (mixed pathology) [16].

A common clinical method to identify AD, AD-CVD, and VaD cases is using the Hachinski ischemic score (HIS) [7,17]. A change in score range cut off for AD to 3 rather than 4 in HIS, i.e., modified HIS, improved the classification accuracy to 78.8% (from 73.3%) when AD was compared to a combined population of VaD and AD-CVD [18]. Another scale, the National Institute of Neurological Disorders and Stroke–Association Internationale pour la Recherche et l’Enseignement en Neurosciences (NINDS-AIREN) scale, identifies VaD (but not AD-CVD) cases more accurately compared to HIS by including the imaging results, which is the main limitation of HIS [6]. Nevertheless and to the best of our knowledge, no highly accurate separation of AD from AD-CVD (alone and not pooled with VaD) has been presented [19,20,21]. Presumably, mixed Alzheimer’s, including AD-CVD, is considered as a category for underdiagnosed cases, and its diagnosis has an important clinical and prognostic value [16,19,22,23].

Electrovestibulography (EVestG), a non-invasive technique that detects vestibulo-acoustic neural activities [24], has shown promising results in the identification of various neurodegenerative [25,26], vertiginous [27,28,29], and neuropsychiatric disorders [30]. Given the extensive direct and indirect links between neuropathologies associated with dementia and the vestibular system [31,32], the distinct impact of AD and AD-CVD has been investigated using EVestG data [31,33]. Analysis of EVestG signals in response to some of its stimuli, as well as using the Montreal Cognitive Assessment (MoCA) score as one of the features, showed >80% accuracy in separating AD from AD-CVD and/or from age-matched healthy controls in our previous studies [31,34]. However, EVestG signals of the entire stimuli were not investigated [31]. Additionally, the time interval histogram (IH) of the detected field potentials (*FPs*), in addition to the average of spontaneous and driven vestibular *FPs* (*FP_ave_*), were not considered in the previous studies [34]. In this study, the potential of EVestG for identifying AD, AD-CVD, and healthy control populations was investigated using both *FP* and IH characteristic curves of the EVestG signal in response to the entire stimuli. A novel hierarchy diagnostic algorithm based on the binary classification outcomes of its lower levels was developed and evaluated.

Low frequency range (proximal to 10 Hz) modulations of IH signals are hypothesized to occur in response to vestibular efferent or α band activity [35]. Spontaneous vestibular efferent activity is seen at 10–50 spikes/s [36] and the α band range is 8–13 Hz. As the experimental average detected gap between every two *FPs* is ~3.3 ms, a 33 *FP* gap approximately corresponds to about 100 ms (10 Hz). Thus, the normalized histogram of the time intervals between each 33 *FPs* (called the IH33 signal) could be of value to investigate for features. EVestG studies showed that the IH33 signal is shifted over the range of frequency depending on the pathology [25,35]. 

Furthermore, imaging studies showed AD biomarkers at an early stage are associated with decreased gamma-aminobutyric acid (GABA) interneurons signaling rather than cholinergic or glutamatergic dysfunction, i.e., due to Aβ and, particularly, Aβ oligomers (AβO) pathology [23,37,38,39,40]. Given that GABA could act as a facilitator in the spontaneous and driven discharge of the vestibular afferents, decreased GABAergic inhibitory function may lead to the defacilitation of/reduction in afferent discharges [41,42]. Thus, an AD feature sensitive to changes in the afferents’ firing pattern could be investigated. Accordingly, the IH33 curve of the AD population is speculated to shift to the lower frequency range or longer time intervals. 

Conversely, studies have shown that the decrease in the cerebrovascular blood flow in animals and humans significantly increases the neuronal inhibition and GABAergic activity [43,44]. This was suggested as a mechanism to reduce the cell injury and enhance the tolerance of neurons to the ischemic and hypoxic condition [45]. Increased synaptic inhibition promotes synchrony of spiking among interneurons and between groups of excitatory neurons [42,46], while it also reduces slow timescale activity in a large population of neurons [47,48,49]. Moreover, a reduction in the blood flow to the vestibular periphery as a result of CVD leads to excitation of the vestibular nuclei, and via the efferent feedback loop, results in modulatory excitation of the vestibular afferents [50]. Based on these findings, we hypothesize that the IH33 curve of the AD-CVD population will shift to the higher frequency range or shorter time intervals compared to that of the AD group. 

The main contribution of this paper is developing a novel hierarchy diagnostic algorithm based on the binary classification outcomes using unbiased features of the IH33 and *FP_ave_* curves of selected EVestG stimuli. While this work is conceptually similar to our previous studies, here we propose a general hierarchical diagnostic algorithm for the separation of AD, AD-CVD, and controls using features of EVestG signals selected through an unbiased selection without any prior assumption(s).

## 2. Materials and Methods

### 2.1. Participants

EVestG data were collected either from the participants who were enrolled in one of the two clinical trial studies for monitoring and treatment of different types of dementia, or from healthy volunteers. From these, data of 16 individuals with AD, 13 with AD-CVD, along with data of 24 healthy controls, which were used in our previous study [34], were adopted for feature extraction, feature selection, and building the classification model. Additionally, the new data of 12 individuals with AD, 9 with AD-CVD, and a maximum of 8 healthy controls were acquired and used as a blind testing dataset. The healthy participants were carefully selected to be free of any significant cerebrovascular disease symptomology, particularly when compared to the AD-CVD population. Thus, two control participants were excluded from the test dataset versus the AD-CVD population (due to having focal neurologic signs).

All participants signed a consent form approved by the Biomedical Research Ethics Board of University of Manitoba prior to being enrolled in the study. The dementia subtype diagnosis was determined by medical specialists (neurologists and neuropsychiatrists) through several visits using clinical assessments and brain imaging results, i.e., magnetic resonance imaging (MRI) and/or positron emission tomography. All the diagnosed AD-CVD individuals also met the NINDS-AIREN criteria for “AD with CVD” [6]. All the diagnosed individuals were assessed based on the modified HIS [17,18], similar to our recent studies [31,34], using their full diagnostic reports (Table 1).

The participants went through a screening hearing test, MoCA [51], and Montgomery–Asberg Depression Rating Scale (MADRS) [52] before EVestG recording. Table 1 lists the participants’ demographics. Except for one healthy control participant with a moderate MADRS score (22 out of 60), none of the participants had any significant depression at the time of testing.

### 2.2. Electrovestibulography (EVestG)

**Figure 1:**
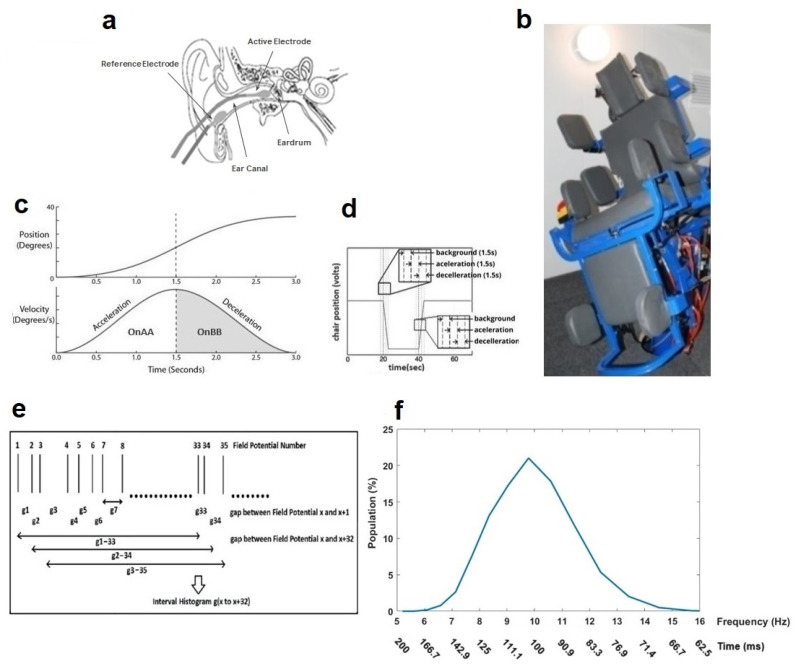
Electrovestibulography (EVestG) recording system and frequency response plot generated by the interval histogram of every 33rd detected field potential (IH33). (**a**) Active and reference electrode placement. (**b**) Hydraulic chair inside the anechoic room. (**c**) Chair position and velocity profiles during movement. (**d**) Chair entire movement pattern. (**e**) IH33 generation process. (**f**) A typical normalized IH33 (time = 1/f).

The detailed methodology for EVestG recordings has previously been presented in [24]. In brief, gelled wick cotton wool tip active and reference electrodes are placed bilaterally proximal to the tympanic membrane and on the outer ear canal, respectively (Figure 1a). A common electrode is placed on the forehead. While seated on a hydraulic chair (Figure 1b) inside an anechoic chamber, in a relaxed state and with eyes closed, the participant’s ears signals are recorded in response to seven different tilting stimuli (Figure 1c) as follows: (a) 15 cm up/downward translation, while the participant is either in the upright sitting position (up/down tilt) or in the supine position (supine up/down tilt); (b) 40-degree rotation to the right side, either in the upright sitting position (rotation tilt) or in the supine position (supine rotation tilt); (c) 40-degree back/forward tilting in the upright sitting position (back/forward tilt); and (d) 40-degree tilting to the right side in the upright sitting position (ipsilateral right and contralateral left tilts), back to the center, and then 40-degree tilting to the left side in the upright sitting position (ipsilateral left and contralateral right tilts). The ipsilateral and contralateral tilts are abbreviated as IT and CT. 

In each tilt, the chair returns to the center before starting another tilt. In every tilt, the chair movement has stationary (background or BGi), acceleration (OnAA), and deceleration (OnBB) phases that each take 1.5 s (Figure 1d). Corresponding to these phases and in each tilt, six 1.5 s segments of recorded EVestG signal are selected for each right/left ear as BGi, OnAA, OnBB, return to center (RTC) BGi, RTC OnAA, and RTC OnBB segments. The selected segments are analyzed offline via the Neural Event Extraction Routine (NEER V5.1) program [24], which detects and averages spontaneous and driven vestibular *FPs* to produce *FP_ave_.* It also detects the time of occurrence of each *FP* and generates a normalized time interval histogram based on every 33rd detected *FP* (Figure 1e), i.e., ~100 ms time interval, named as IH33 (Figure 1f), during both static and dynamic conditions. It consists of 25 logarithmically spaced bins spanning the time interval or the frequency range (f = 1/time). The IH33 signal is used to focus on the low-frequency modulation of the *FPs*’ firing pattern proximal to 10 Hz, which is hypothesized to be linked to the alpha band [35] and the lower end of vestibular efferent activity [36]. All the recordings were carried out at the EVestG lab, Riverview Health Center, Winnipeg, Manitoba, Canada. As the discriminative features in relation to the *FP_ave_* signal were already selected in our previous study [34], here we explain the IH33 feature selection procedure and then use of the final selected features to develop the hierarchy diagnostic algorithm.

The noisy IH33 signals that occurred due to muscle artifacts, poor electrode placement, or jittery movement of the chair were checked manually and removed from the analysis (approximately 5% of the IH33 signals). Typically, IH33 signals corresponding to the following conditions were excluded if: (i) the registered times of occurrence of the detected *FPs* did not produce a smooth curve versus the *FPs*’ number (similar to a stepwise rather than a semi-linear curve); (ii) the number of registered *FPs* was less than 350 or the times of occurrence of *FPs* spanned below 97% of the recorded segment duration (i.e., below 1.46 s compared to 1.5 s); or (iii) the shape of the IH33 signal looked like a bimodal histogram rather than a normal one with the smaller peak exceeding more than 10 percent of the population. 

### 2.3. Signal Analysis

**Figure 2:**
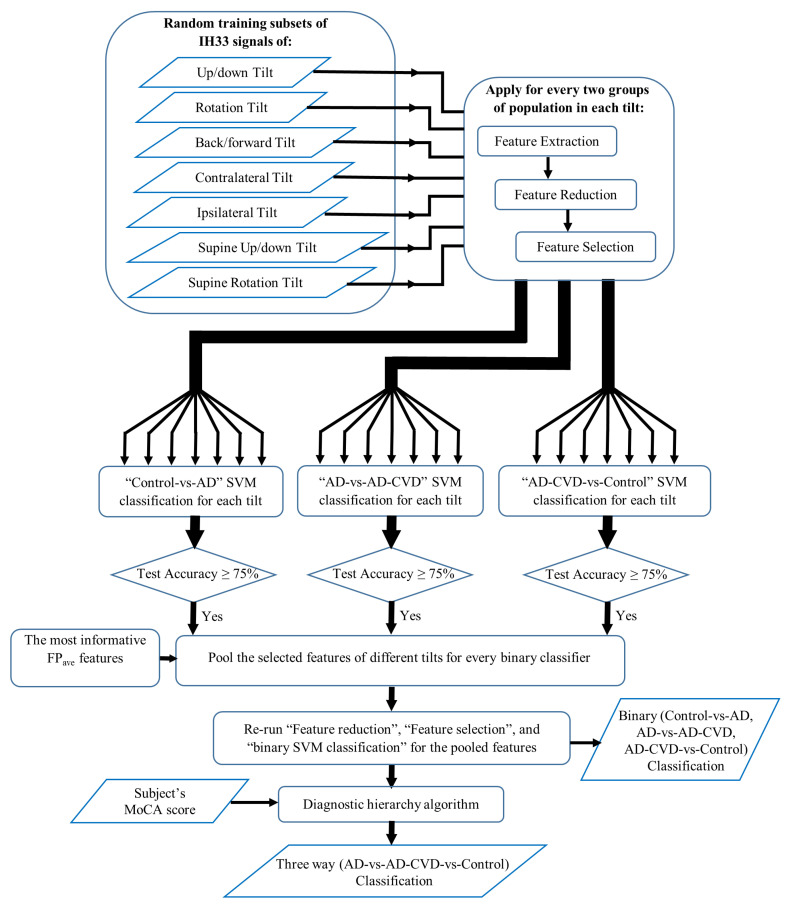
A summary of the proposed approach for classification.

Figure 2 demonstrates a summary of the proposed approach for classification. The IH33 signals from every tilt were analyzed separately. Each tilt included IH33 signals of the six aforementioned segments for each (left/right) ear. Moreover, IH33 signals of the background segments (BGi or RTC BGi) of each ear, which were either in the upright sitting position (7 segments) or in the supine position (4 segments), were averaged to be used in the upright sitting or the supine tilt, respectively. These IH33 signals are named as “Upright average” and “Supine average” IH33 signals. Additionally, summation and subtraction (asymmetry) of the left and right ear BGi or RTC BGi IH33 signals were included in the analysis (“LR” and “L-R” were added to the label for summation and subtraction, respectively).

Having data of seven different tilts from three populations (AD, AD-CVD, and Control), an unbiased feature extraction method, similar to our previous study (for a one-vs.-one classification approach [34]) was conducted. Thus, 21 binary classifiers, i.e., seven Control-vs.-AD, seven AD-vs.-AD-CVD, and seven AD-CVD-vs.-Control classifiers, were designed. The procedure for each binary classification is presented below. 

### 2.4. Feature Extraction

To extract characteristic unbiased features from IH33 signals, subsets of the training data were selected as training sets by randomly leaving 20% of the training data of every population out for testing. For binary classification, the minimum number of selected training sets for which all of the training data were used in a “left-out” set at least once was equal to 25 (5 × 5). Considering the small training dataset and to improve the stability of the outcome features, the number of random training sets was chosen as 1600 (40 × 40). In each training set, the standard error bands around the averaged IH33 signals of the two groups were searched for any mutual separation (i.e., the separation occurred if the lower standard error band of one group had higher values compared to the upper standard error band of the other group in time/frequency bins). In case of separation, and thus moving the standard error bands of the averaged IH33 signals of the two groups away from each other, two possible time/frequency regions at either side of the crossing of the two averaged IH33 signals were identified. The feature was computed as the average values of the bins of one region subtracted from those of the other region to magnify the shift in the IH33 signals. It is worth noting that the values of the first and last two bins, as well as the three bins corresponding to the peak value of the IH33 average signal of the two groups, were excluded as they were susceptible to noise (due to insignificant large differences in variance). Then, based on the normality test result calculated by the Shapiro–Wilk Normality test [53], either the non-parametric Wilcoxon–Mann –Whitney test [54] or the Unpaired *t*-test [55] was applied on the feature. If a feature was found to be significant (*p*-value < 0.05), it was saved as a selected feature in the training set. As the number of extracted features in each training set was large, feature reduction and selection were performed similar to the approach in [34] and summarized as follows: 

### 2.5. Feature Reduction and Selection

In each training set and after imputation of the missing values, feature reduction was performed based on selecting (maximum of three) feature combinations, which resulted in the highest classification accuracy using supervised support vector machine (SVM) classification [56] in an exhaustive search scheme. In cases where the feature sets had the same classification accuracy, the feature set with the lowest number of missing values was selected (please find the detailed information of feature reduction in the Supplementary File of [34]). Assuming the first and second classes in a binary classification as the positive and negative classes, respectively, the classification accuracy in a binary classification was calculated as follows:

(1) $$Accuracy=\frac{TP+TN}{TP+FP+TN+FN}$$

where *TP*, *TN*, *FP*, and *FN* are true positive, true negative, false positive, and false negative cases, respectively. 

Using the reduced feature set in every training set, a supervised 10-fold cross-validation SVM classification was applied and the averaged training and testing performances were calculated. Then, the feature set that yielded the highest test accuracy and its features that were the most frequently repeated ones among the selected features across all the training sets was selected. Since the identified region(s) of the IH33 signal for the repeated features varied due to difference in the training set, the region(s) that was present for more than 50% of the repetitions, herein named as the common region(s), was selected to be used in the final classification. Given that the total number of possible training sets was larger than what was generated, the procedures of feature extraction, reduction, and selection were repeated three times with different random training sets to test if similar final features were selected. This stage ensures that the number of shuffles of the training data (training sets) is enough to be representative of the entire training dataset and to prevent overfitting of the classification model.

### 2.6. Binary Classification

The selected features were recalculated based on their common region(s), and missing data were imputed for the entire dataset. The features were Z-score normalized before and after imputation. Then, a 10-fold cross-validation SVM classification was applied and the averaged training and testing performances were calculated. In every binary classification (Control-vs.-AD, AD-vs.-AD-CVD, or AD-CVD-vs.-Control), the tilts for which their selected features yielded ≥75% averaged test accuracy were chosen as the most informative ones in relation to using the IH33 signal in that classification. 

In order to find the most informative features among the top IH33 and *FP_ave_* selected features across all the tilts, the IH33 features of the most informative tilt(s) were pooled with the top *FP_ave_* selected features of our previous work [34]. Then, the above feature reduction, selection, and classification were applied on the entire pooled features. It is noteworthy that, at this stage, the features of the training sets were known; thus, no feature extraction was needed. The most informative selected features of each classifier were then used in a 5-fold (as the blind test set was smaller) cross-validation SVM classification for the blind testing dataset and the averaged performances were computed. 

### 2.7. Diagnostic Hierarchy Algorithm

Given the three SVM binary classifiers and using the approximated posterior probabilities of an SVM model via the Platt scaling method [57], six probabilities were calculated for each participant. Every two of these probabilities identified the extent to which a participant belonged to either of the two groups out of the three populations, i.e., Control (C), AD, or AD-CVD. Additionally, the averaged sensitivity and specificity of the binary classifiers on the training data were incorporated as a weighting coefficient to the above probabilities. This helps in accounting for the binary classifier that had a higher classification result. Then, the weighted averages of the two probabilities for each group were calculated and used as a score that showed the degree of assignment of a participant to that group. Finally, the three scores for every participant (score of being identified as AD, AD-CVD, and C) were normalized to represent a probability measure. As an example, the following formulas show the calculation of the normalized score (probability measure) of a participant as a control subject:

(2) $${Score}_{C}=Average\{P_{C_{C-vs-AD}}\times {W_{C}}_{c-vs-AD}+P_{C_{AD-CVD-vs-C}}\times {W_{C}}_{AD-CVD-vs-C}\}$$

(3) $${Normalized\;Score}_{C}={Score}_{C}/\left({Score}_{C}+{Score}_{AD}+{Score}_{AD-CVD}\right)$$

where $P_{C_{C-vs-AD}}$ and $\;P_{C_{AD-CVD-vs-C}}$ are the probabilities of a participant to be identified as a control in the “Control-vs.-AD” and “AD-CVD vs. Control” classifiers, respectively. In addition, ${W_{C}}_{c-vs-AD}$ is the averaged sensitivity of the “Control-vs.-AD” binary classifier, and ${W_{C}}_{AD-CVD-vs-C}$ is the averaged specificity of the “AD-CVD vs. Control” binary classifier. The sensitivity and specificity of the binary classifiers were calculated as follows:

(4) $$Sensitivity=\frac{TP}{TP+FN}$$

(5) $$Specificity=\frac{TN}{TN+FP}$$

**Figure 3:**
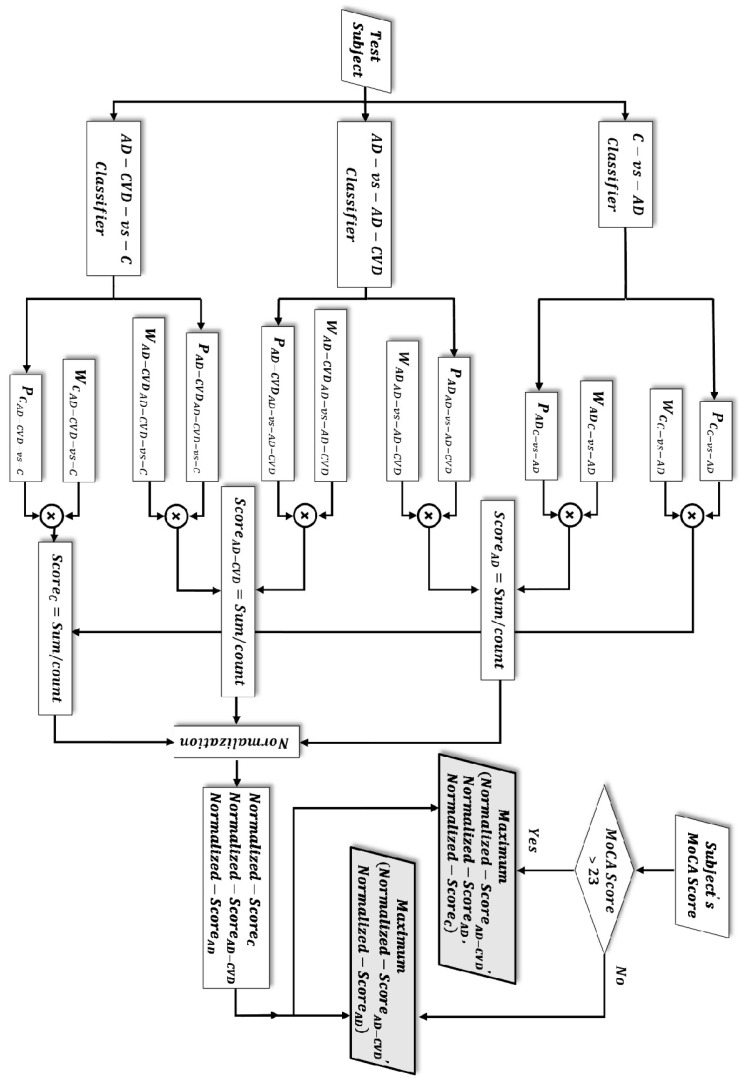
Flow chart of the three-way classification. Depending on the subject’s Montreal Cognitive Assessment (MoCA) score, either of the two grey-color-filled parallelograms determines the classification result. The test subject is classified to the population group in which it achieved a higher/highest normalized score.

Moreover, the MoCA score was used (as in [31]) to increase the three-way classification accuracy by separating healthy cognitive aging from a spectrum of cognitively impaired participants (control versus patient). A recent meta-analysis revealed that a MoCA cutoff score of 23 lowers the false positive rate (i.e., falsely identifying a participant as a cognitively impaired individual) and shows an overall better diagnostic accuracy [58]. Consequently, if a participant’s MoCA score was 23 or below, which implies the participant’s cognitive impairment, the participant was classified to either the AD or AD-CVD group depending on which of the two normalized scores was higher. On the other hand, participants with MoCA scores above 23 were classified to one of the three groups (Control, AD, or AD-CVD), based on which of their computed normalized scores was the highest. Figure 3 shows the flow chart of the diagnostic hierarchy algorithm for the three-way classification. The final selected features and classification performances are reported in the Results section.

### 2.8. Statistical Analysis

One-way multivariate analysis of covariance (MANCOVA) was conducted on the final selected most informative features of the Control-vs.-AD, AD-CVD-vs.-Control, and AD-vs.-AD-CVD classifiers with sex and age as covariates. All of the signal processing steps were performed using the MATLAB (v2017a) environment except for the analyses of covariance, which were performed using SPSS v21 (IBM, New York, NY, USA).

## 3. Results

Table 2 lists the averaged test binary classification performances of the Control-vs.-AD, AD-vs.-AD-CVD, and AD-CVD-vs.-Control classifiers on the entire training dataset for every tilt. In each classification, the tilts are sorted based on the averaged classification accuracy. This table shows that back/forward, supine up/down, and up/down tilts in the AD-vs.-AD-CVD classifier, supine up/down and IT tilts in the AD-CVD-vs.-Control classifier, and supine up/down tilt in the Control-vs.-AD classifier are selected as the most informative tilts (≥75% accuracy) in the classification of their corresponding populations.

Considering the IH33 selected features of the most informative tilts and pooling them with the most informative *FP_ave_* selected features that were previously identified in [34], the final selected most informative features were found. A set of three *FP_ave_* features that were selected across all the tilts for the AD-vs.-AD-CVD classifier in [34] showed 78% averaged test accuracy; thus, these features were pooled with the IH33 features in the AD-vs.-AD-CVD classifier. Table 3 presents the final selected most informative features for the three binary classifications. In this table, the selected features are listed based on the EVestG tilt, the type of signal (*FP_ave_* or IH33), the EVestG segment, and the recorded ear side. The area under the curve (AUC) values associated to the receiver operating characteristic (ROC) curves of the 10-fold cross-validation for each feature was calculated and averaged. This denotes the relevance of each feature to the target class. As seen, the signal type of all of the final selected features was found to be the IH33 signal. Moreover, the majority of the final features (six out of nine) were selected from the supine up/down tilt recording.

Table 4 reports the averaged test performance of the binary classifiers on the blind testing dataset. The AUC values associated with the ROC curves of the 5-fold cross-validation for each feature were calculated and averaged. As seen, the averaged AUC calculated values for the blind testing dataset were close to the averaged AUC values for the training dataset. Moreover, among the three classifiers, AD-vs.-AD-CVD achieved the highest accuracy (80.9%). 

**Figure 4:**
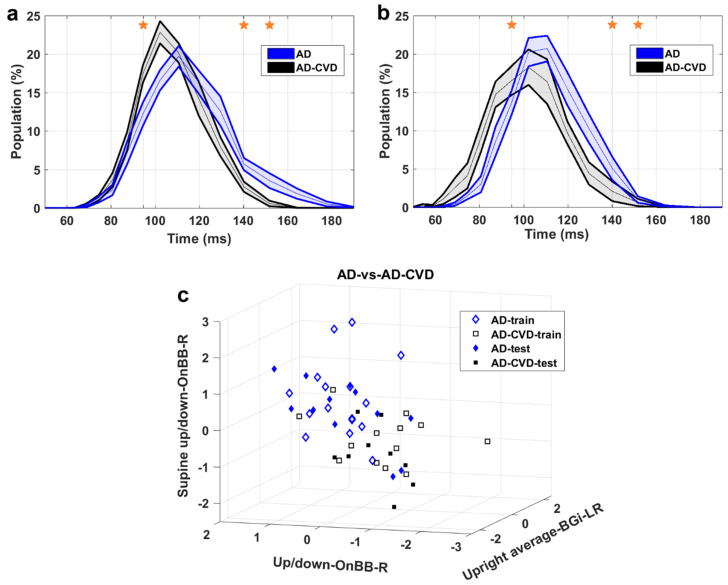
AD-vs.-AD-CVD classification. (**a**) IH33 signals of the final selected most informative feature of AD-vs.-AD-CVD classification that achieved the highest averaged AUC for the training dataset, i.e., supine up/down−OnBB−R, for training and (**b**) blind test datasets. Mean with standard error band is shown for ease of visualization. The middle point of time bins that contributed to the calculation of the feature are marked with stars and are as follows: 94.5, 140.2, 151.8 ms. (**c**) The AD-vs.-AD-CVD classification scatter plot of the features of Table 3 for training and blind testing datasets.

**Figure 5:**
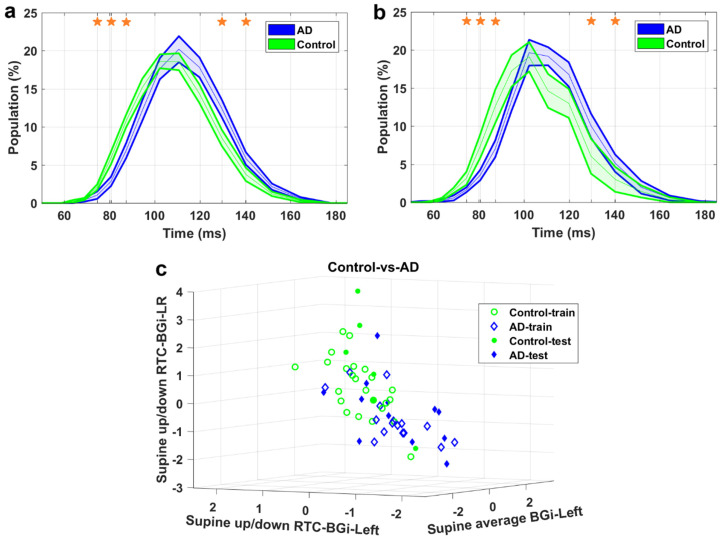
Control-vs.-AD classification. (**a**) IH33 signals of the final selected most informative feature of Control-vs.-AD classification that achieved the highest averaged AUC for the training dataset, i.e., supine up/down-RTC-BGi-LR, for training and (**b**) blind test datasets. Mean with standard error band is shown for ease of visualization. The middle point of time bins that contributed to the calculation of the feature are marked with stars and are as follows: 74.5, 80.6, 87.3, 129.6, and 140.2 ms. (**c**) The Control-vs.-AD classification scatter plot of the features of Table 3 for training and blind testing datasets.

**Figure 6:**
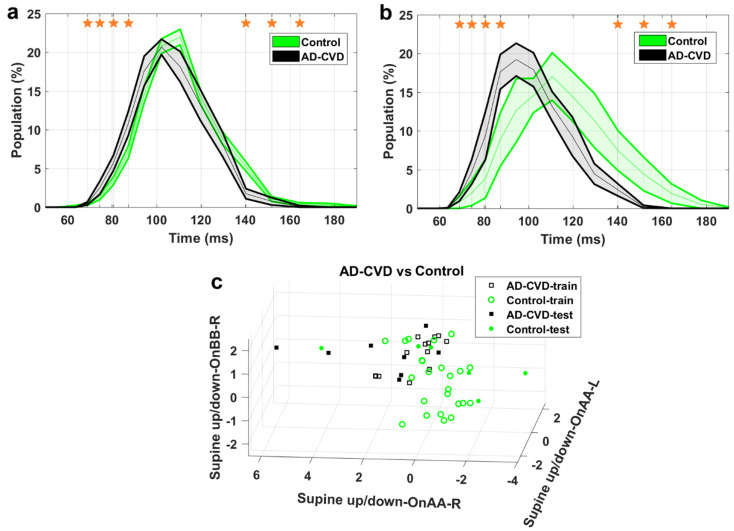
AD-CVD-vs.-Control classification. (**a**) IH33 signals of the final selected most informative feature of AD-CVD-vs.-Control classification that achieved the highest averaged AUC for the training dataset, i.e., supine up/down-OnAA-R, for training and (**b**) blind test datasets. Mean with standard error band is shown for ease of visualization. The middle point of time bins that contributed to the calculation of the feature are marked with stars and are as follows: 68.9, 74.5, 80.6, 87.3, 140.2, 151.8, and 164.3 ms. (**c**) The AD-CVD-vs.-Control classification scatter plot of the features of Table 3 for training and blind testing datasets.

Figure 4, Figure 5 and Figure 6 demonstrate the IH33 signals of the final selected most informative features that achieved the highest averaged AUC for the training dataset in every binary classification. These signals are plotted separately for the training and blind testing datasets. The time bins that contributed to the calculation of the significant feature are mentioned and shown with a star in each Figure. The classification scatter plots of the features of Table 3 are also presented for training and blind testing.

As seen in the Figures, the averaged AD IH33 signal is shifted towards longer time intervals/lower frequencies, i.e., a larger population percentage of firing in lower frequencies as well as a smaller population percentage of firing in higher frequencies, compared to those of the control and AD-CVD IH33 signals. Conversely, the averaged AD-CVD IH33 signal is shifted towards shorter time intervals/higher frequencies, i.e., a larger population percentage of firing in higher frequencies as well as a smaller population percentage of firing in lower frequencies, compared to those of the control and AD IH33 signals. 

Table 5 shows the three-way classification performance including the confusion matrix, one versus rest approach sensitivity and specificity (i.e., one population is assumed as the positive group and the other two populations are merged together as the negative group), and balanced accuracy for the training and blind testing datasets. Balanced accuracy is calculated as the arithmetic mean of the sensitivities or recalls for each class; thus, it naturally provides a higher weight to the classes with a smaller sample size, which can be more appropriate if the classes are not exactly balanced. Thus, balanced accuracies of 85.7%, and 79.6% were attained on the training and blind testing datasets, respectively.

### Statistical Analysis

MANCOVA was applied on the combined selected features of the Control-vs.-AD, AD-CVD-vs.-Control, and AD-vs.-AD-CVD classifiers. A statistically significant difference was found between the two populations after accounting for sex and age; no significant effect was found for sex or age covariates (details are provided in the Supplementary File).

## 4. Discussion

In this pilot study, we applied our developed automated algorithm [34] to extract unbiased features of EVestG IH33 signals in regard to the separation of pairs of AD, AD-CVD, and healthy control populations. We designed three binary classifiers for every EVestG tilt and compared the accuracies of classification across different EVestG tilts. According to Table 2, the supine up/down tilt was selected as one of the most informative stimuli (achieved an accuracy of ≥75% when applied alone) in all of the three binary classifiers, while the back/forward and up/down (sitting position) tilts, and the IT tilt were selected in the AD-vs.-AD-CVD and AD-CVD-vs.-C classifications, respectively. It is noteworthy to mention that although the IT tilt achieved ≥ 75% accuracy in AD-CVD-vs.-C classification, it was not very successful in the identification of AD-CVD participants (specificity = 55%). Among the EVestG tilts, the supine up/down tilt predominantly stimulates the utricular organ, and together with the sitting up/down tilt, which mainly stimulates the saccule, contains the lowest contribution of muscle artefacts, hemodynamic effects, and participant anxiety. Considering the closeness of the utricular maculae to the stapes and thus to the EVestG recording electrode, it is more likely that the EVestG response is mostly driven from the utricle [59]. Therefore, the selection of the supine up/down tilt for mutual separation of the three aforementioned groups can be considered physiologically and experimentally reasonable. According to epidemiological human studies, saccular and utricular impairments are associated with five- and four-fold increased odds of AD, respectively [60]. Human studies on measuring Cervical Vestibular Evoked Myogenic Potential and MRI analysis have suggested that decreased saccular function is significantly related to a lower average hippocampal volume [61,62]. These results may give a picture of the cognitive impairment impact of AD on the otolithic organ, particularly the saccule, thus justifying the selection of the up/down tilts for AD-vs.-AD-CVD classification. Finally, the back/forward tilt also showed a high AD-vs.-AD-CVD classification accuracy. Features of this tilt together with the supine up/down tilt were previously found to be discriminative in the prediction of the response to rTMS treatment for AD and AD-CVD participants [33]. Although the back/forward tilt stimulates almost the entire vestibular organ, it could contain blood pressure change and anxiety components, which both need to be carefully studied. It is noteworthy that the back/forward tilt features were not selected as the final selected most informative features in our study. 

Using the combination of IH33 features from the selected most informative tilts and the previously selected *FP_ave_* features [34], the final selected most informative EVestG features in the classification of pairs of AD, AD-CVD, and control populations were identified. According to Table 3, all of the selected features were found from IH33 signals, and as expected and hypothesized, they were from either the supine up/down or up/down tilts. It is worth noting that the discriminative features that were selected as being predictive of rTMS efficacy in our previous study were also found from IH33 signals. The final selected most informative feature with the highest averaged AUC from the training dataset (0.79) for separation of the AD and AD-CVD populations was found from the upward moving deceleration (OnBB) segment of the supine up/down tilt (Figure 4). Interestingly, the same feature was selected previously [31] but more intuitively for both classification and prediction of the response to treatment in AD and AD-CVD populations. Furthermore, the final selected most informative features of each classifier were used to classify the blind testing dataset. The moderate averaged performances in Table 4 show the robustness of the extracted features. According to Figure 4, Figure 5 and Figure 6, the averaged AD IH33 signals corresponding to the final selected most informative features were shifted towards lower frequencies, i.e., a larger population percentage of firing in lower frequencies as well as a smaller population percentage of firing in higher frequencies, compared to those of the control and AD-CVD IH33 signals. On the other hand, the averaged AD-CVD IH33 signals corresponding to the final selected most informative features were shifted towards higher frequencies, i.e., a larger population percentage of firing in higher frequencies as well as a smaller population percentage of firing in lower frequencies compared to those of the control and AD IH33 signals. This trend was consistent between the training and blind testing datasets. 

Synaptic loss, which precedes neurodegeneration, is one of the pathological hallmarks of AD and the strongest predictor of cognitive decline [63,64]. Much evidence indicates that Aβ oligomers (AβO), rather than Aβ plaques, could mediate the neurotoxic effects of the Aβ pathway [63,65,66], as they build up earlier and are more potent than Aβ plaques in eliciting abnormalities in synaptic function and neural network activity [64,65]. Over the past few years, lines of evidence in animal models, and in in vitro and human studies have suggested that synaptic failure, particularly at the early stage of AD, is induced by neuronal hyperactivity rather than later stage hypoactivity [64,67,68,69]. They support the major role of AβO accumulation in neuronal hyperactivity observed at the onset of AD, in both cortical and subcortical brain regions, although other AD-peptides may also contribute [40,64,68,70]. 

In the past decades, studies have implicated the disruption of cholinergic and glutamatergic neurotransmission in instigating synaptic failure and AD pathology [23]. However, an increasing number of studies support the onset of AD being linked to the decrease in GABAergic inhibitory function as a result of the pathological elevation of AβO peptides [39,40]. This in turn can induce activation of the excitatory glutamatergic response and cause a vicious cycle of an excessive release of Aβ as a result of the disruption of the excitatory/inhibitory neuronal balance [40,64]. Given the GABAergic inhibitory role in regulating, synchronizing, and preventing excess neuronal signaling [23,71], it is not surprising that GABAergic-decreased inhibition increases the incidence of neuronal firing in local assemblies of interconnected neurons in the early stage of AD [23,39,63]. However, this enhanced activity occurs locally among the proportion of neurons that are more vulnerable and not the overall neuronal network [40,67]. Therefore, despite the increased local hyperactivity and due to the lack of unified synchrony of larger assemblies of interconnected neural circuits involving different brain regions, the pathologically elevated AβO in AD could result in network activity destabilization, reduced excitatory current, and synaptic depression [63]. As evidence, this localized neuronal hyperactivity causes gamma wave conductance disruption (lower power of gamma oscillatory activity) in the MCI and early stage AD pathology [39,64,72]. This may imply the lack of overall brain wave modulation of higher frequency firing during the onset of AD. 

Studies have shown a similar yet lower degree of various GABAergic component alterations, including depression of GABA levels [39], increased GAD activity [37], synaptic function disruption at GABAergic terminals [37], and increased sensitivity of GABA receptors [73], indicating the lack of inhibitory responses in subcortical regions such as the thalamus, Locus Coeruleus (LC), cochlear, and vestibular nucleus compared to cortical regions during the AD pathology or aged brain. Notably, AβO in the LC neurons of AD patients showed a close association with impaired GABA A receptors, which result in the defacilitation of overall neural network activity due to local (at single cell levels) neuronal hyperexcitability [65]. Given the LC bidirectional links to the vestibular nucleus [74], and similar GABAergic alternations such as the increased sensitivity of GABAergic receptors in an aged vestibular nucleus complex [73], this may imply that AβO-induced GABAergic inhibitory disruption may reduce the facilitation of vestibular firing, particularly afferent discharges, at the vestibular periphery, thus resulting in the speculation about a lower frequency firing pattern for AD patients. 

On the contrary, it has been shown in animal and human studies that, as a result of a decrease in the blood flow supply of the brain tissues, the neuronal inhibition and GABAergic activity significantly increases [43,44] and then decreases during the recovery process. Moreover, the increase in GABA levels is observed in patients with vascular risk factors (diabetic aged participants that were compared to controls) [75,76]. Similarly, GABA levels are shown to increase after inhibiting brain glycogen in Type 2 diabetic rats [77]. It is argued that the increased GABA activity could be assumed to be an underlying mechanism that reduces cell injury by antagonizing glutamate excitotoxicity, enhances the tolerance of neurons to the ischemic and hypoxic condition, and has significant neuroprotective effects [45]. Given that the inhibition increases fast spike synchrony between excitatory neurons [42,46], and reduces the slow (long) timescale relationship among large population of neurons [47,48,49], it is probable that, as a result of a chronic CVD condition, the synchrony of the neuronal network in the transmission of faster firing increases and leads to a firing pattern that is shifted towards higher frequencies. Conforming to this could be the excitation of vestibular nuclei and vestibular afferents via the efferent feedback loop following hypotension [50]. 

Finally, a hierarchy diagnostic algorithm was developed for three-way classification by averaging the pairs of probabilities that identified a participant to belong to one of the three population groups. The averaged specificity or sensitivity of the classifiers over the training dataset were also used as weighting coefficients of the probabilities. Thus, three normalized linear weighted average scores were calculated for each participant. Then, the participant’s final diagnosis was the group where he/she had the highest normalized score. This could be similar to the way the brain of a physician concludes a clinical diagnosis: by comparing the symptoms against each class of dementia (and healthy controls) and going with the one with the highest likelihood of probability.

As shown in our previous studies [31], the averaged IH33 signal of the control population sits in between the AD and AD-CVD ones (a graph of the IH33 signals for the three populations is added in the Supplementary File). This causes averaging of the probabilities that assign a participant to either the AD or AD-CVD group to sometimes be misleading. As an example, an AD participant can gain a low classification probability of being a control in the Control-vs.-AD classifier; however, due to the special placement of the IH33 signals of the three populations over the range of frequency (or time), the same participant may gain a high classification probability of being a control in the AD-CVD-vs.-Control classifier. Thus, the average probability of being a control may become large, which is not correct. We solved this issue by incorporating a cutoff MoCA score, as a preprocessing step before EVestG signal classifications, which separated the cognitively impaired participants (MoCA ≤ 23) from the healthy ones. The groupings of such participants were later identified by comparing only the AD and AD-CVD scores of the three-way classifier. 

## 5. Conclusions

In this pilot study, we extracted the most informative features of the EVestG signals to classify pairs of AD, AD-CVD, and healthy control populations in an unbiased and automated manner. We also identified the EVestG tilts for which their extracted features were the best candidates for the above separations. Additionally, the robustness of the most informative features was tested via a blind testing dataset. Using the participants’ MoCA score and the normalized linear weighted average score of the binary classifiers, we developed a novel diagnostic algorithm for a three-way classification that resulted in 85.7% and 79.6% accuracy in the training and blind testing datasets, respectively. The possible physiological changes support the selected EVestG features. Disruptions to inhibition associated with GABAergic activity might be responsible for the shift of AD/AD-CVD EVestG IH33 signals to lower/higher frequencies.

## 6. Limitations and the Future of the Study

One of the limitations of this study is the small sample size of the dataset. Given the difficulties of participant recruitment, particularly participants who are diagnosed at the early stage of AD or AD-CVD, and the chance of not being able to record some participants’ EVestG signals due to excessive ear wax, a slow data collection process and small dataset were the result. Moreover, noise corrupted signals due to artefactual reasons, which could have led to missing data and a further reduction in the sample size. Considering the heterogeneity of biological data, a larger sample size could represent the entire population more accurately; hence, the reliability and credibility of the selected features could be enhanced as well. Additionally, a larger sample size may include patients who suffer from AD mixed with other types of dementia, i.e., AD-non-specific, as not all mixed AD patients are AD-CVD. The hierarchical algorithm that is introduced in this study may have the potential to be generalized to separate AD-non-specific groups from the AD, AD-CVD, and control groups. The discriminative features can also be used in future studies to monitor and investigate the effects of interventions, and to predict the disease’s progression.

## Figures and Tables

**Table 1:** Control, Alzheimer’s disease (AD), and AD mixed with levels of cerebrovascular disease (CVD) symptomology (AD-CVD) study participants’ demographics.

	Age (µ ± SD)	Sex	MoCA (µ ± SD)	Modified HIS (µ ± SD)	MADRS (µ ± SD)
Control, N = 24	65.3 ± 7	9 M	27.6 ± 1.7	-	2.6 ± 5.7
AD, N = 16	72.5 ± 7.5	11 M	16.4 ± 4.8	1.8 ± 1.2	1.9 ± 2.8
AD-CVD, N = 13	75.8 ± 7.3	9 M	17 ± 4.4	5.6 ± 1.4	3.1 ± 4
Blind testing AD, N = 12	67.2 ± 7.1	9 M	16 ± 6.7	1.3 ± 1.3	4.7 ± 4.7
Blind testing AD-CVD, N = 9	71.3 ± 7.7	6 M	16.8 ± 6.7	4.6 ± 1	2.2 ± 3.6
Blind testing controls:					
- N = 8 used vs. AD	69.4 ± 5	4 M	26 ± 2.5	-	4 ± 3.4
- N = 6 used vs. AD-CVD	69.8 ± 4.1	3 M	27 ± 1.8	-	3 ± 3.2

(µ ± SD) values represent mean ± standard deviation.

**Table 2:** Supervised support vector machine (SVM) binary classification averaged test results on the entire dataset.

Averaged Test Performances of the Binary Classifiers on Training Dataset											
AD-vs.-AD-CVD				Control-vs.-AD				AD-CVD-vs.-Control			
Tilt	Sens (%)	Spec (%)	Acc (%)	Tilt	Sens (%)	Spec (%)	Acc (%)	Tilt	Sens (%)	Spec (%)	Acc (%)
Back/forward ^a^	95	60	80	Supine up/down ^a^	86.7	80	82.3	Supine up/down ^a^	60	90	80.3
Supine up/down ^a^	70	80	76.7	Back/forward	75	70	73	IT ^a^	55	88.3	77
Up/down ^a^	80	70	75	Supine rotation	80	60	70.3	Rotation	45	85	72.1
IT	85	60	74.2	Up/down	86.7	45	69.7	Back/forward	30	88.3	68.3
CT	70	60	65.8	IT	76.7	55	68	Supine rotation	25	86.7	65.5
Supine rotation	80	40	62.5	CT	83.3	35	63.5	Up/down	10	85	59
Rotation	80	40	61.7	Rotation	75	25	54	CT	15	76.6	54.8

The results are sorted according to the highest average accuracy. ^a^ The tilts that achieved an accuracy ≥ 75% are marked as the most informative tilts. Sens: sensitivity, Spec: specificity, and Acc: accuracy.

**Table 3:** The final selected most informative features (F1, F2, and F3) for the three binary classifications.

Selected Most Informative Features of the Binary Classifiers				
	Tilt	Signal Type	Segment_Side	AUC
AD-vs.-AD-CVD	F1—Upright average	IH33	BGi_LR	0.64
	F2—Up/down	IH33	OnBB_R	0.77
	F3—Supine up/down	IH33	OnBB_R	0.79
Control-vs.-AD	F1—Supine average	IH33	BGi_L	0.62
	F2—Supine up/down	IH33	RTC_BGi_L	0.78
	F3—Supine up/down	IH33	RTC_BGi_LR	0.82
AD-CVD-vs.-Control	F1—Supine up/down	IH33	OnAA_L	0.51
	F2—Supine up/down	IH33	OnAA_R	0.78
	F3—Supine up/down	IH33	OnBB_R	0.51

The selected features are listed based on the EVestG tilt, the type of signal, i.e., averaged field potentials (*FP_ave_*) or IH33, the EVestG segment, the recorded ear side, i.e., left (L), right (R), or summation of left and right (LR) sides, and the averaged area under the curve (AUC) values associated with the receiver operating characteristic (ROC) curves of 10-fold cross-validation.

**Table 4:** SVM binary classification averaged test results on the blind testing dataset.

Averaged Test Performances of the Binary Classifiers on the Blind Testing Dataset				
	Sens (%)	Spec (%)	Acc (%)	AUC
AD-vs.-AD-CVD	75.11	88.9	80.9	F1 = 0.66, F2 = 0.77, F3 = 0.79
Control-vs.-AD	87.6	66.4	74.9	F1 = 0.62, F2 = 0.8, F3 = 0.82
AD-CVD-vs.-Control	72.5	67	70.2	F1 = 0.5, F2 = 0.77, F3 = 0.51

Sens: sensitivity, Spec: specificity, and Acc: accuracy. F1, F2, and F3 are the selected features of each binary classifier according to Table 3.

**Table 5:** Three-way classification averaged training and blind testing results.

Train, Test Dataset Classification Results	True Class						
Total Number = 54, 27		**AD**	**AD-CVD**	**Control**	**Sens vs. Rest (%)**	**Spec vs. Rest (%)**	**Balanced Accuracy (%)**
Predicted Class	AD	15, 10	2, 0	2, 0	93.8, 83.3	89.2, 100	85.7, 79.6
	AD-CVD	1, 2	11, 8	3, 2	84.6, 88.9	90, 77.8	
	Control	0, 0	0, 1	19, 4	79.2, 66.7	100, 95.2	

The confusion matrix, one versus rest approach sensitivity, specificity, and balanced accuracy for the training and blind testing datasets are listed. Sens: sensitivity and Spec: specificity.

## Data Availability

The data presented in this study are available from the corresponding author upon reasonable request. For any data sharing, one must contact NeuralDX Pty. Ltd. (Toorak, VIC, Australia).

## Supplementary Materials

The following supporting information can be downloaded at: https://www.mdpi.com/article/10.3390/medicina59122091/s1, Figure S1: IH33 signals of the average of left and right RTC BGi segments in Supine Up//down tilt (Supine Up/down-RTC-BGi-LR) for the three populations over the range of frequency or time.; Table S1: One-way MANCOVA on the selected features of C-vs.-AD, AD-CVD-vs.-C, and AD-vs.-AD-CVD classifiers controlling for age and sex. Each feature is named based on the IH33 that is extracted from in terms of the IH33′s tilt name, segment (seg), and the ear side, left (L) or right (R). Refs [78,79,80] are in the file.

## Abbreviations

Aβ	Amyloid-β
AβO	Amyloid-β oligomers
Acc	Accuracy
AD	Alzheimer’s disease
AD-CVD	AD mixed with levels of cerebrovascular disease symptomology
AUC	Area under the curve
BGi	Background segment
C	Control
CT	Contralateral tilt
CVD	Cerebrovascular disease
EVestG	Electrovestibulography
FP	Field potential
FP_ave_	Average of spontaneous and driven vestibular field potentials
GABA	Gamma-aminobutyric acid
HIS	Hachinski ischemic score
IH	Interval histogram
IH33	33-Interval histogram
IT	Ipsilateral tilt
L	left
LC	Locus Coeruleus
LR	Summation of left and right signals
L-R	Subtraction of left and right signals
µ	Mean
MADRS	Montgomery–Asberg Depression Rating Scale
MANCOVA	Multivariate analysis of covariance
MoCA	Montreal Cognitive Assessment
MRI	Magnetic resonance imaging
NEER	Neural Event Extraction Routine
NINDS-AIREN	National Institute of Neurological Disorders and Stroke–Association Internationale pour la Recherche et l’Enseignement en Neurosciences
OnAA	Acceleration segment
OnBB	Deceleration segment
R	right
ROC	Receiver operating characteristic
RTC	Return to center
SD	Standard deviation
Sens	Sensitivity
Spec	Specificity
SVM	Supervised support vector machine
VaD	Vascular dementia
VN	Vestibular nucleus
