# Supplementary material for: Evaluating the Diagnostic Value of Electrovestibulography (EVestG) in Alzheimer’s Patients with Mixed Pathology: A Pilot Study
Source: Medicina (Kaunas). 2023 Nov 28;59(12):2091. doi: 10.3390/medicina59122091 (PMC10744488; doi:10.3390/medicina59122091)
Supplement: Supplementary file 1 [file medicina-59-02091-s001.zip › medicina-2671804-supplementary.pdf]

medicina

MDPI

Article

Evaluating the Diagnostic Value of Electrovestibulography (EVestG) in Alzheimer's Patients with Mixed Pathology: A Pilot Study

Zeinab A. Dastgheib, Brian J. Lithgow and Zahra K. Moussavi *

Diagnostic and Neurological Processing Research Laboratory, Biomedical Engineering Program, University of Manitoba, Riverview Health Centre, Winnipeg, MB R3L 2P4, Canada; zeinab.dastgheib@umanitoba.ca (Z.A.D.); brian.lithgow@umanitoba.ca (B.J.L.)
* Correspondence: zahra.moussavi@umanitoba.ca; Tel.: +1-204-474-7023

Section A

The special placement of the IH33 signals of the three populations over the range of frequency or time (i.e., the control population sits in between AD and AD-CVD ones).

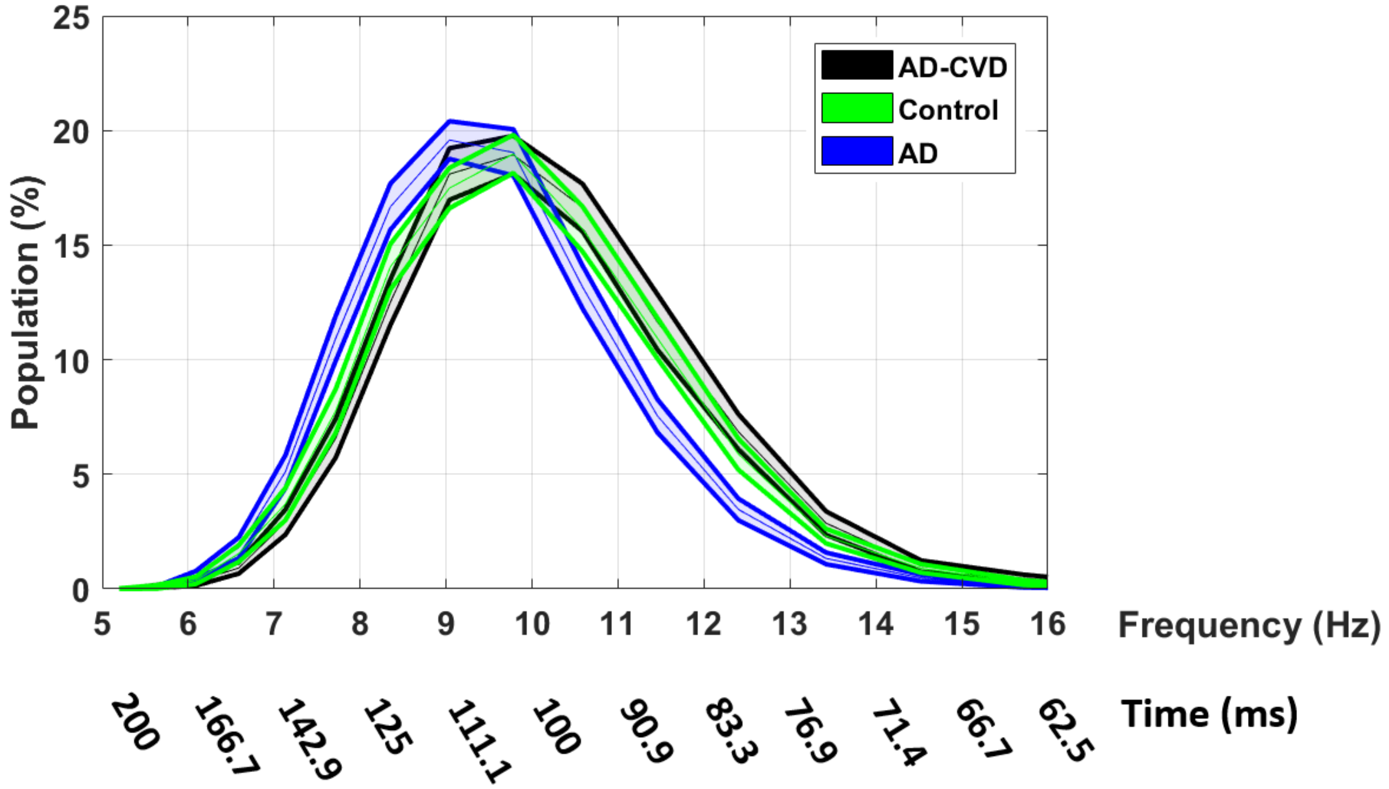

Figure S1. IH33 signals of the average of left and right RTC BGi segments in Supine Up//down tilt (Supine Up/down-RTC-BGi-LR) for the three populations over the range of frequency or time.

Section B

A one-way MANCOVA was applied using the final selected most informative features of Control-vs-AD, AD-CVD-vs-Control, and AD-vs-AD-CVD classifiers with controlling for age, and sex covariates [1, 2]. The first feature in Control-vs-AD and the second and third features in AD-CVD-vs-Control classifier were transformed to have a normal distribution using the method described in [3]. Table S1 summarizes that analysis.

Table S1. One-way MANCOVA on the selected features of C-vs-AD, AD-CVD-vs-C, and AD-vs AD-CVD classifiers controlling for age and sex. Each feature is named based on the IH33 that is extracted from in terms of the IH33's tilt name, segment (seg), and the ear side, left (L) or right (R).

**Main One-way MANCOVA**										
**Selected feature**		**Covariates**	**Covariate p value**	**Wilkes Lambda**	**F**	**MANOVA** p value	**Partial** ◎2	**Power**	**Box's M p value**	**Lavene's p value**
**Multivariate main effect-pathology**										
C-vs-AD	- Supine average BGi_L<br>- Supine up/down RTC_BGi_L<br>- Supine up/down RTC_BGi_LR	age<br>sex	0.693<br>0.698	0.764	F(3,53) =5.457	0.002	0.236	0.921	0.131	0.951<br>0.136<br>0.756
**Univariate main effects**										
- Supine average BGi_L					F(1,55)=15.916	<0.001	0.224	0.975		
- Supine up/down RTC_BGi_L					F(1,55)=10.183	0.002	0.156	0.880		
- Supine up/down RTC_BGi_LR					F(1,55)=5.244	0.026	0.087	0.614		
**Multivariate main effect**										
AD-CVD-vs-C	- Supine up/down_ OnAA_L<br>- Supine up/down_ OnAA_R<br>- Supine up/down_ OnBB_R	age<br>sex	0.889<br>0.835	**0.717**	F(3,45) =5.935	0.002	0.283	0.939	0.081	0.159<br>0.252<br>0.084
**Univariate main effects**										
- Supine up/down_OnAA_L					F(1,47)=6.453	0.014	0.121	0.701		
- Supine up/down_OnAA_R					F(1,47)=12.147	0.001	0.205	0.927		
- Supine up/down_OnBB_R					F(1,47)= 6.451	0.014	0.121	0.701		
**Multivariate main effect-pathology**										

AD-vs-AD-CVD	- Upright average_BGi_LR<br>- Up/down_OnBB_R<br>- Supine up/down_OnBB_R	age<br>sex	0.594<br>0.344	0.719	F(3,43) = 5.59	0.003	0.281	0.922	0.302	0.297<br>0.687<br>0.596

Univariate main effects

- Upright average_BGi_LR	F(1,45)= 7.215	0.01	0.138	0.748
- Up/down_OnBB_R	F(1,45)= 8.939	0.005	0.166	0.833
- Supine up/down_OnBB_R	F(1,45)= 9.978	0.003	0.181	0.871

References

1. Landau S, Everitt B; A handbook of statistical analyses using SPSS. Chapman & Hall/CRC, Boca Raton, **2004**
2. Pituch KA, Stevens J, Applied multivariate statistics for the social sciences: analyses with SAS and IBM's SPSS, 6th edition. Routledge/Taylor & Francis Group, New York, **2016**
3. Templeton, G.F. A Two-Step Approach for Transforming Continuous Variables to Normal: Implications and Recommendations for IS Research. *Commun. Assoc. Inf. Syst.* **2011**, *28*, 41–58, https://doi.org/10.17705/1cais.02804.
